# In vitro–in vivo Validation of Stimulatory Effect of Oat Ingredients on Lactobacilli

**DOI:** 10.3390/pathogens10020235

**Published:** 2021-02-19

**Authors:** Cindy Duysburgh, Pieter Van den Abbeele, Alison Kamil, Lisa Fleige, Peter John De Chavez, YiFang Chu, Wiley Barton, Orla O’Sullivan, Paul D. Cotter, Karina Quilter, Susan A. Joyce, Mike Murphy, Gillian DunnGalvin, Timothy G Dinan, Massimo Marzorati

**Affiliations:** 1 ProDigest BV, 9052 Ghent, Belgium; Cindy.Duysburgh@prodigest.eu (C.D.); pieter@prodigest.eu (P.V.d.A.); 2 PepsiCo R&D, Barrington, IL 60010, USA; Alison.Kamil@pepsico.com (A.K.); lisa.fleige@pepsico.com (L.F.); Peter.DeChavez@pepsico.com (P.J.D.C.); YiFang.Chu@pepsico.com (Y.C.); 3 Teagasc Food Research Centre, Moorepark Fermoy, P61 C996 Cork, Ireland; wiley.barton@teagasc.ie (W.B.); orla.osullivan@teagasc.ie (O.O.); Paul.Cotter@teagasc.ie (P.D.C.); 4 APC Microbiome Institute, T12 Cork, Ireland; K.Quilter@ucc.ie (K.Q.); s.joyce@ucc.ie (S.A.J.); t.dinan@ucc.ie (T.G.D.); 5 School of Biochemistry and Cell Biology, University College Cork, T12 Cork, Ireland; 6 Atlantia Food Clinical Trials, Heron House Offices, First Floor, Blackpool, T23 Cork, Ireland; mmurphy@atlantiafoodtrials.com (M.M.); gdunngalvin@atlantiafoodtrials.com (G.D.); 7 Department of Psychiatry and Neurobehavioural Science, University College Cork, T12 Cork, Ireland; 8 Center of Microbial Ecology and Technology (CMET), Ghent University, 9000 Ghent, Belgium

**Keywords:** *Bifidobacterium*, *Lactobacillus*, beta-glucan, intestine, microbiota, in vitro–in vivo correlation

## Abstract

The prebiotic activity of a commercially available oat product and a novel oat ingredient, at similar β-glucan loads, was tested using a validated in vitro gut model (M-SHIME^®^). The novel oat ingredient was tested further at lower β-glucan loads in vitro, while the commercially available oat product was assessed in a randomised, single-blind, placebo-controlled, and cross-over human study. Both approaches focused on healthy individuals with mild hypercholesterolemia. In vitro analysis revealed that both oat products strongly stimulated *Lactobacillaceae* and *Bifidobacteriaceae* in the intestinal lumen and the simulated mucus layer, and corresponded with enhanced levels of acetate and lactate with cross-feeding interactions leading to an associated increase in propionate and butyrate production. The in vitro prebiotic activity of the novel oat ingredient remained at lower β-glucan levels, indicating the prebiotic potential of the novel oat product. Finally, the stimulation of *Lactobacillus* spp. was confirmed during the in vivo trial, where lactobacilli abundance significantly increased in the overall population at the end of the intervention period with the commercially available oat product relative to the control product, indicating the power of in vitro gut models in predicting in vivo response of the microbial community to dietary modulation.

## 1. Introduction

Prebiotics are defined as non-digestible substrates that are selectively utilised by the gut microbiome, thereby conferring beneficial effects for the host [1]. Oat products contain several soluble components capable of exerting prebiotic properties, such as β-glucans, arabinoxylans, arabinogalactans, resistant starch, and polyphenolic compounds. However, many of the health-promoting effects related to the consumption of oat products have been attributed to the intake of β-glucans. These non-starch polysaccharides consist of β-linked chains of D-glucose monomers, differing in their branching structure, viscosity, solubility, and molecular weight [2], and they have been associated with a reduction of systemic cholesterol levels [3], regulation of blood glucose concentrations, improved weight management [4], and modulation of immune function [2].

In order to exert their health-related properties, β-glucans need to be released from the cell wall of the oat groats, a process which is highly affected by the applied processing techniques. Indeed, hydrothermal processing has been linked with reduced release of oat β-glucans [5]. Mechanical processing, on the other hand, results in increased extractability of β-glucans from the oat groats, probably by increasing the exposed surface area [6]. Recently, Van den Abbeele et al. [7] reported an association between the β-glucan content and the prebiotic potential of different oat ingredients that were produced by different mechanical processing techniques, suggesting that different processing techniques might impact the potential functional properties of the final oat ingredients by affecting β-glucan extractability.

Several human studies have explored the prebiotic activity of oat products [8,9,10,11]. Connolly et al. [11], for instance, investigated the prebiotic activity of whole-grain oat granola, containing 1.3g β -glucan per dose in a human population with mild to moderate hypercholesterolemia. It was shown that intake of whole-grain oat granola selectively enhanced the abundance of *Bifidobacterium* and *Lactobacillus* species and total bacterial population compared to baseline levels, accompanied by reduced systemic levels of total and LDL cholesterol. Indeed, numerous studies have linked the consumption of oat products with hypocholesterolaemic effects [12,13,14]. While their cholesterol-lowering activity has been associated with increased excretion of bile acids and cholesterol in faeces due to the enhancement of viscosity of the intestinal content [15], recent research suggests a potential role of the gut microbial community [16]. Indeed, modulation of the intestinal microbiota might impact bile acid metabolism by stimulating bacterial bile salt hydrolase activity of certain bacterial groups, thereby affecting cholesterol excretion [17,18]. Furthermore, microbial production of propionate has been associated with a reduction of cholesterol levels [19,20]. As several in vitro and in vivo studies have reported modulation of the gut microbiota upon oat supplementation [11,21,22], a potential mechanism of action of oat products in controlling cholesterol homeostasis might be related to its prebiotic properties.

Assessments of the prebiotic properties of dietary fibres are often performed during in vitro studies. In vitro approaches offer an appropriate alternative to clinical trials because interactions between the gut microbial community and dietary fibres can be investigated at the site of action by strict control of environmental parameters [23,24]. 

As the prebiotic properties of oat ingredients might be involved in the lowering of cholesterol levels, the initial aim of this study was to compare the prebiotic activity of a commercially available oat product and a novel oat ingredient, standardised to provide similar levels of β-glucan, in a model of the human gastrointestinal tract of different healthy individuals with elevated cholesterol levels, making use of the validated Mucosal Simulator of the Human Intestinal Microbial Ecosystem (M-SHIME^®^, ProDigest and Ghent University, Ghent, Belgium) [25], in order to analyse potential similarity in prebiotic response of the microbial community following oat consumption. Moreover, it was assessed if the potential prebiotic properties of the novel oat product remained at lower β-glucan levels in terms of effects on microbial metabolic activity and community composition of the luminal and mucosal gut microbiome in vitro. Finally, a clinical trial, with a corresponding human population, was carried out with the commercially available oat product in order to determine if in vitro observations translated in vivo to provide evidence that the use of in vitro gut models can be used to predict dietary outcomes following supplementation of novel ingredients in vivo.

## 2. Results

### 2.1. Altered Microbial Metabolic Activity in Response to Oat Treatment in vitro

**Figure 1:**
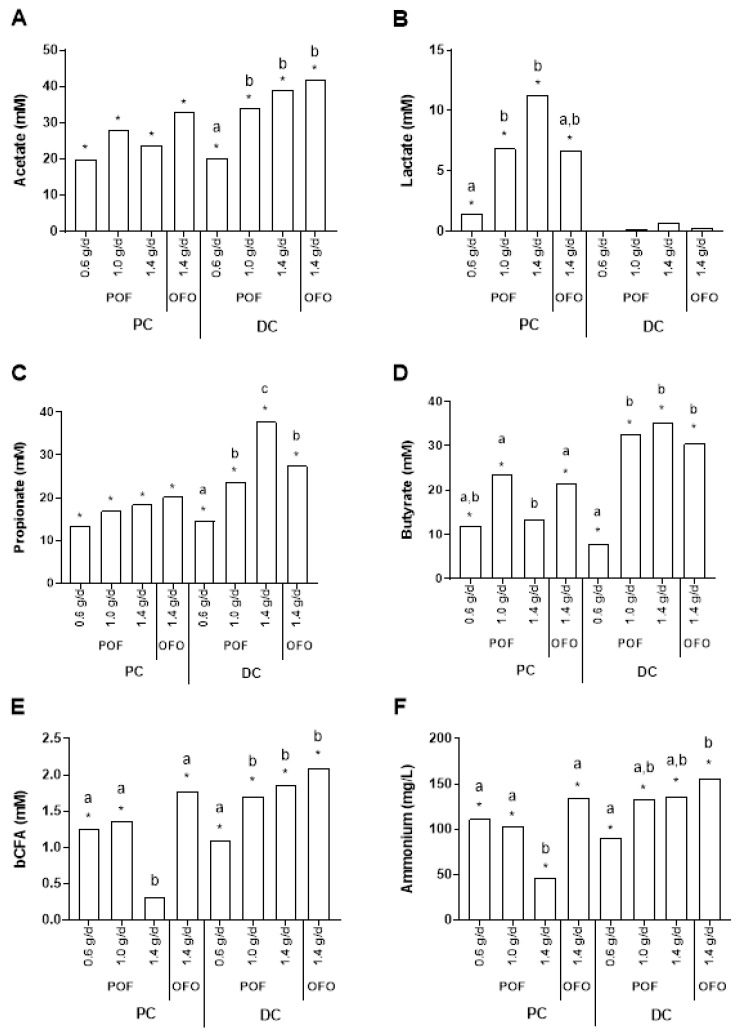
Effects on primary microbial metabolites. The average increase in (**A**) acetate (mM), (**B**) lactate (mM), (**C**) propionate (mM), (**D**) butyrate (mM), and (**E**) branched-chain fatty acid (bCFA; mM) and (**F**) ammonium (mg/L) levels during the treatment period as compared to the control period upon treatment with old-fashioned oats (OFO) at a dose of 1.4 g/d and pre-cooked oat flour (POF) at a dose of 0.6 g/d, 1.0 g/d, and 1.4 g/d in the proximal (PC) and distal colon (DC) of the human gastrointestinal tract for three human donors tested. For optimal observation of consistent effects over the different donors tested, the average of the three donors is presented (*n* = 3). Statistically significant differences relative to the control period are indicated with *, whereas statistically significant differences between the different test conditions, in the proximal and distal colon, respectively, are indicated with different letters (*p* < 0.05).

The short-chain fatty acids (SCFAs) detected consisted mainly of acetate, propionate, and butyrate (Figure 1) and trace amounts of branched-chain fatty acid (bCFA) (Figure 1). First, all test conditions significantly increased acetate (*p* < 0.0001), propionate (*p* < 0.0001), and butyrate (*p* < 0.0001) levels in both colon regions as compared to the control period. The only exception was noted in the proximal (PC) upon supplementation of pre-cooked oat flour (POF) at a concentration of 1.4 g β-glucan/day, where a marked, but not statistically significant (*p* = 0.211), increase in butyrate levels was still observed (+13.2 mM). The latter could be due to the fact that lactate most strongly accumulated (Figure 1) for this condition (i.e., an average increase in lactate levels of 11.3 mM). While acetate and propionate levels were not significantly different as a consequence of the supplementation of old-fashioned oats (OFO) relative to POF at a concentration of 1.4 g β-glucan/day in the PC, butyrate levels were significantly higher upon treatment with OFO in the PC (*p* < 0.0001), with an additional average increase of 8.3 mM upon OFO fermentation as compared to POF. In the DC, no significant differences in acetate and butyrate production were observed between supplementation of OFO and POF at a concentration of 1.4g β-glucan/day, whereas POF more strongly enhanced propionate production as compared to OFO (i.e., on average an additional increase of 10.2 mM) in this colonic region (*p* = 0.007). With respect to the different doses of POF, acetate, propionate, and butyrate levels increased in a dose-dependent manner in the distal colon (DC), with the highest final SCFA levels observed upon supplementation of the highest concentration of POF. 

In terms of lactate production (Figure 1), a significant increase in lactate levels was observed in the PC in response to the different treatments, whereas lactate levels remained around the detection limit in the DC. In both colon regions, lactate levels were not significantly different between OFO and POF dosed at a concentration of 1.4 g β-glucan/day. Furthermore, lactate levels increased in a dose-dependent manner in the PC for the different doses of POF, with the highest lactate levels being observed upon supplementing the highest dose of POF.

With respect to markers of proteolytic fermentation (Figure 1), all treatments significantly increased ammonium and bCFA levels in response to the treatment in both colon regions, except for bCFA levels in the PC upon supplementation of POF at a concentration of 1.4 g β-glucan/day (*p* = 0.279). Overall, significantly lower ammonium and bCFA levels were observed in the PC for POF versus OFO at a concentration of 1.4 g β-glucan/day (*p* < 0.0001). Furthermore, in the PC, supplementation of POF at the highest concentration tested resulted in the lowest production of bCFA and ammonium, indicating the presence of a dose-response effect. In the DC, an opposite trend was observed. 

### 2.2. Altered Microbial Composition in Response to Oat Treatment in vitro

To compare the prebiotic properties of the different test products and conditions, qPCR analysis of specific health-related groups (*Bifidobacterium* spp. and *Lactobacillus* spp.) was performed (Table 1). First, luminal *Lactobacillus* levels increased significantly in both colon regions upon treatment with the different test products. Overall, the strongest increase was observed upon supplementation of POF at a concentration of 1.4 g β-glucan/day, resulting in significantly higher *Lactobacillus* levels as compared to the other test conditions (only POF at a concentration of 1.0 g β-glucan/day resulted in similar increases in the luminal DC). Similar effects were observed for *Bifidobacterium* levels, which significantly increased in the luminal environment of both colon regions upon treatment with all the different test products. It was noted that OFO more strongly stimulated *Bifidobacterium* levels than POF at a concentration of 1.4 g β-glucan/day in the PC. Overall, effects on mucosal microbes (Table 1) were similar to the changes reported for the luminal microbiota, even though less pronounced, showing that the test products were able to stimulate the luminal and the mucosal levels of bifidobacteria and lactobacilli.

**Figure 2:**
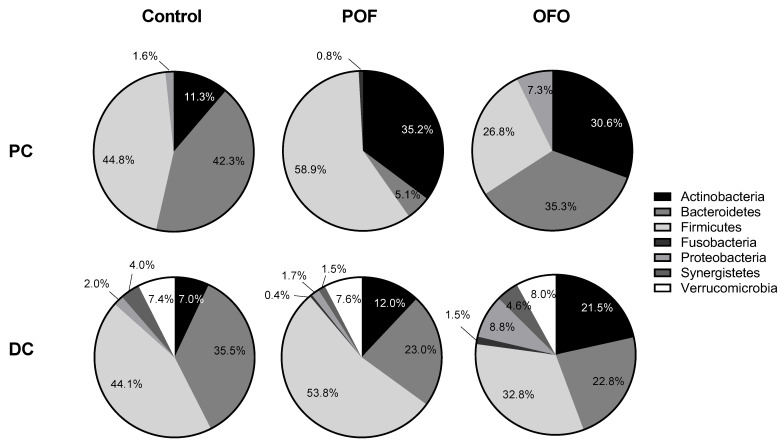
Microbial community composition as assessed via 16S-targeted Illumina sequencing. Abundance (%) at microbial phylum level in the luminal environment of the proximal (PC) and distal colon (DC) of the human gastrointestinal tract at the end of the control (C; *n* = 6/donor) and the treatment (TR; *n* = 3/donor) period upon treatment with OFO at a dose of 1.4 g/d and POF at a dose of 1.4 g/d for three human donors tested. For optimal observation of consistent effects over the different donors tested, the average of the three donors is presented (*n* = 3).

In order to investigate more extensively the effects of treatment with OFO and POF at a dose of 1.4 g β-glucan per day on microbial community composition, microbiome profiling of the luminal PC and DC compartment was performed using 16S-targeted Illumina sequencing. At phylum level (Figure 2), it was noted that at the main site of fermentation, i.e., the lumen of the PC, both test products strongly increased Actinobacteria levels. Similar observations were made in the DC. Additionally, a consistent increase in Firmicutes levels was observed upon treatment with POF in the PC and DC, while supplementation of OFO resulted in enhanced Proteobacteria levels. 

At the family level, the primary focus was on the treatment effects of POF and OFO, at a concentration of 1.4 g β-glucan/day, at the main site of fermentation, i.e., the lumen of the PC (Table 2). For the luminal DC (Table 3), similar observations were made and therefore only specific and distinct changes versus the PC referred to here. First, treatment with POF and OFO strongly increased *Bifidobacteriaceae* levels (*p* < 0.0001). At the operational taxonomic unit (OTU) level, this was mainly attributed to a significant increase in *Bifidobacteriaceae* OTU 1 (related to *Bifidobacterium adolescentis*). To a lesser extent, *Bifidobacteriaceae* OTU 41 (related to *Bifidobacterium bifidum*) and *Bifidobacteriaceae* OTU 47 (related to *Bifidobacterium longum*) were stimulated. A significant increase in the abundance of *Lactobacillaceae* was observed upon treatment with both test products, with the strongest effects for POF supplementation. The increased abundance of *Lactobacillaceae* was mainly attributed to the increased abundance of *Lactobacillaceae* OTU 5 (related to *Pediococcus acidilactici*). Furthermore, upon treatment with both test products, *Enterococcaceae, Enterobacteriaceae* (*p* < 0.0001), and *Prevotellaceae* were significantly enriched in the luminal PC, while *Akkermansiaceae* (*p* = 0.0031 for POF and *p* = 0.0118 for OFO) and *Enterobacteriaceae* (*p* < 0.0001) were enhanced in the luminal DC. For the latter bacterial family, treatment with OFO resulted in a stronger enrichment as compared to POF supplementation in both the PC (*p* < 0.0001) and DC (*p* < 0.0001). For POF, an increased abundance of *Veillonellaceae* was observed upon treatment (*p* < 0.0001 in PC and *p* = 0.0007 in DC), which was mainly attributed to an increase in *Veillonellaceae* OTU 22 (related to *Veillonella parvula*) and *Veillonellaceae* OTU 11 (related to *Veillonella ratti*). Furthermore, treatment with POF consistently decreased *Bacteroidaceae* (*p* < 0.0001) and *Lachnospiraceae* (*p* < 0.0001) levels in the luminal PC, resulting in significantly (*p* < 0.0001) lower levels as compared to treatment with OFO. Treatment with OFO, on the other hand, resulted in increased *Acidaminococcaceae* levels in the PC, while in the DC, *Bacteroidaceae* levels decreased (*p* = 0.0063) at the expense of *Prevotellaceae* (*p* = 0.0019). Finally, the butyrate-producing *Ruminococcaceae* family was stimulated significantly in the PC environment upon treatment with the different test products, while decreased abundance was observed in the DC (*p* = 0.0009 for POF and *p* < 0.0001 for OFO). 

### 2.3. In vitro–in vivo Comparison of Microbial Response to OFO Treatment

**Figure 3:**
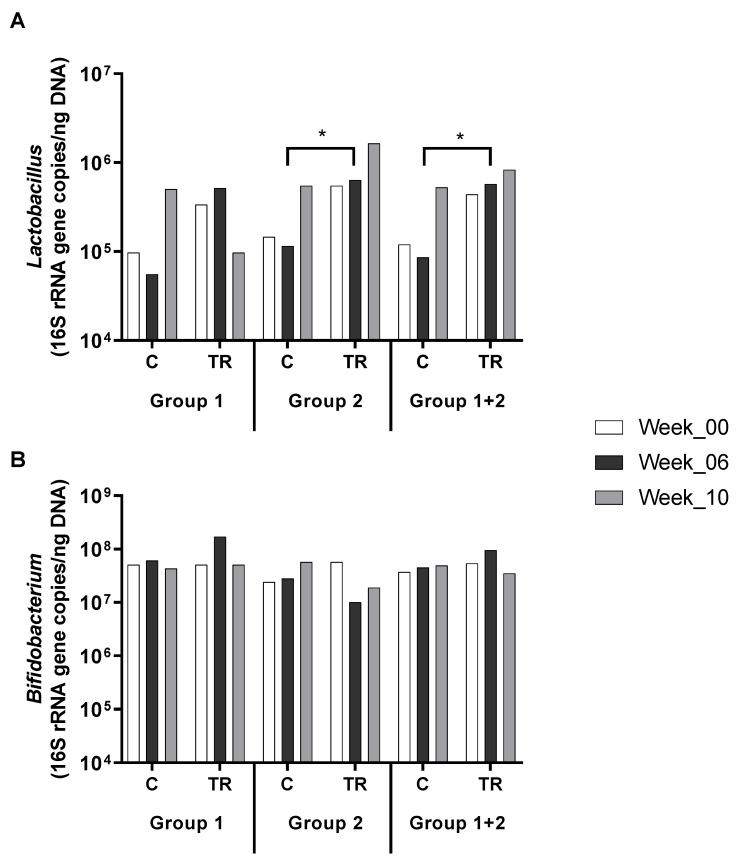
Effects on microbial community composition in vivo. Effect of intervention with OFO (TR) on faecal (**A**) *Lactobacillus* and (**B**) *Bifidobacterium* levels as compared to an intervention with the control test product (C) during a randomised, single-blind, cross-over study in healthy individuals with elevated cholesterol levels for the per protocol (PP) population, with samples taken at baseline (week_00), at the end of the intervention period (week_06) and at the end of the washout period (week_10). Results are presented for Group 1 (received OFO during the first intervention period), Group 2 (received OFO during the second intervention period), and the overall (Group 1 + 2) population. Results are presented as mean (16S rRNA gene copies/ng DNA). * indicates statistically significant differences between control and treatment within a specific experimental week for Group 1, Group 2, and Group 1 + 2 (*p* < 0.05).

In vitro, the prebiotic activity of OFO was characterised by a significant stimulation of lactobacilli and bifidobacteria in both the luminal and mucosal environment (Table 1). Therefore, selective primers were selected to analyse the levels of *Lactobacillus* spp. and *Bifidobacterium* spp. in the faecal samples collected during the in vivo trial by means of quantitative polymerase chain reaction (qPCR). During the in vivo trial, stimulation of *Lactobacillus* spp. was observed (Figure 3A). Indeed, significant stimulation of *Lactobacillus* species was observed at the end of the intervention period with OFO as compared to the control test product for the overall population (*p* = 0.037) and in the population designated as ‘Group 2’, i.e., the group that received the investigational product during the second intervention period (*p* = 0.017; Figure 3A). For *Bifidobacterium* levels (Figure 3B), no overall significant differences were observed between intervention with OFO and the control test product, although a trend towards increased *Bifidobacterium* levels was observed upon intervention with OFO in the ‘Group 1’ population (i.e., the group that received the investigational product during the first intervention period) (*p* = 0.299). With respect to plasma SCFA levels, no statistically significant differences were observed between intervention with OFO and the control test product.

## 3. Discussion

In the present study, the potential prebiotic effects of prolonged administration of the commercially available oat product OFO were assessed in the human gastrointestinal tract of different healthy individuals with mild hypercholesterolemia. For this purpose, the validated in vitro M-SHIME^®^ model [25] was utilised, allowing analysis of an intestinal microbial community that is fully stable prior to treatment [26]. It followed that the prebiotic activity of OFO was characterised by strong stimulation of *Lactobacillus* and *Bifidobacterium* species in the intestinal lumen and the simulated mucus layer of the PC and DC. Lactobacilli [27] and bifidobacteria [28] are saccharolytic gut microbes and are both capable of producing high levels of lactic acid, thereby exerting antimicrobial properties [29] and stimulating trophic interactions with other bacteria resulting in the production of secondary metabolites such as butyric acid [30]. Due to their saccharolytic metabolism, *Lactobacillus* and *Bifidobacterium* species mainly thrive in the proximal regions of the colon [31]. Therefore, the effects of prebiotic compounds on these bacterial groups are often more difficult to observe in in vivo trials. However, during the current study, the stimulation of *Lactobacillus* spp. by OFO was also observed in the faecal samples of human subjects with mild hypercholesterolemia during the randomised, single-blind, placebo-controlled, cross-over study. This is a striking observation as in vivo studies are inherently confounded by large variability due to interindividual differences among individuals and a strong influence of external factors such as diet [32]. Moreover, the investigated in vivo samples considered faecal samples that are only to a limited extent representative for the microbial changes in the PC. For *Bifidobacterium* levels, no significant differences were observed between intervention with OFO and the control test product, although in the population that received OFO during the first intervention period a trend towards increased *Bifidobacterium* levels was observed upon intervention with OFO. Overall, existing scientific evidence and the preliminary results in this study show the significant stimulatory effects of OFO on lactobacilli in human subjects with mild hypercholesterolemia, which points to the prebiotic potential of OFO. Because it has been reported extensively that lactobacilli and bifidobacteria exert hypocholesterolaemic effects both in both animals and humans [33,34,35,36], further in vivo studies are warranted to explore this effect for OFO. Furthermore, as stimulation of *Lactobacillus* spp. by OFO in vivo correlated with the obtained in vitro data, the current study shows that in vitro gut models might be an interesting tool in predicting in vivo response of the microbial community to dietary modulation. 

The in vitro component of the current study also aimed to assess the prebiotic potential of a novel oat product, i.e., POF, relative to OFO. It was revealed that both exerted a highly similar prebiotic activity. First, both test products resulted in significantly higher levels of acetate and lactate, which correlated with the strongly enhanced levels of *Lactobacillaceae* and *Bifidobacteriacea* species [31], indicating the involvement of these bacterial groups in primary substrate degradation upon supplementation of the different oat products. At the OTU level, the main changes were attributed to a significant increase in an OTU related to *Bifidobacterium adolescentis*. It has been previously reported that fermentation of oat bran stimulates the growth of *Bifidobacterium adolescentis* in vitro [37], while also fermentation of β-glucans has been associated with this bacterial species [38]. The significant stimulation of *Lactobacillaceae* upon treatment with the different test products was linked with increased abundance of an OTU related to *Pediococcus acidilactici*, which has been associated with immune-enhancing effects [39]. Furthermore, both test products enhanced *Akkermansiaceae* levels in the DC. The only representative of *Akkermansiaceae* in the gut is the mucin-degrading *Akkermansia muciniphila*, which has been correlated with several health benefits, such as the inverse relationship between colonisation of *Akkermansia muciniphila* and inflammatory conditions [40]. Additionally, significantly enhanced levels of propionate and butyrate were observed upon administration of both test ingredients. Administration of oat products has previously been linked with increased levels of butyrate [41,42]. Indeed, Knudsen et al. [42] reported that addition of oat bran to the diet of pigs increased butyrate concentrations in the luminal environment of the porcine colon. However, administration of β-glucan enriched oat fractions to the porcine diet did not result in butyrate enrichment, indicating that other dietary components (e.g., arabinoxylans) are responsible for the observed increase in butyrate concentration upon oat supplementation. β-glucans on the other hand have been shown to selectively stimulate propionate levels in the colonic environment [7,43,44]. It was proposed that the hypocholesterolaemic effect of oat fibres [12,13,14] might be associated with this propionogenic response. Indeed, upon its production, propionate is transported to the liver, where it impacts cholesterol and fatty acid synthesis [19,20]. Moreover, next to the reduction of cholesterol levels, health-beneficial effects of propionate include weight management by stimulation of satiety [45], regulation of immune function in adipose tissue [46,47], and protection against cancer development [48]. The stimulation of SCFA production by the different oat ingredients therefore suggests their prebiotic potential. 

In addition, some differences were observed between the fermentation of OFO and POF. For instance, supplementation of POF resulted in the strongest stimulation of *Lactobacillus* levels in the PC and DC, while OFO administration more strongly enhanced butyrate production and *Bifidobacterium* concentrations in the PC. Previous in vitro research by Van den Abbeele et al. [7] revealed that product-specific microbial pathways were boosted upon administration of structurally different oat ingredients. For instance, while all products resulted in a significant increase in *Bifidobacterium* levels, oat bran showed the strongest bifidogenic effect of the six oat ingredients investigated, indicating that slight differences in prebiotic response exist among oat-derived products, as observed in the current study.

Moreover, in vitro investigations were carried out to determine if the potential prebiotic properties of the novel oat product POF persisted at lower test doses. It was observed that acetate, propionate, and butyrate levels increased in a dose-dependent manner in the DC with the highest final SCFA levels observed upon supplementation of the highest concentration of POF. However, even at the lowest concentration tested, POF stimulated SCFA production and *Lactobacillus* and *Bifidobacterium* levels, indicating that the prebiotic activity of POF remained at lower β-glucan levels. Overall, the obtained results demonstrate the prebiotic potential of the novel oat product POF, even when administered at lower concentrations. 

In order to further investigate the impact on the luminal microbial community composition upon supplementation of POF and OFO at similar β-glucan load, 16S-targeted Illumina sequencing was performed. Treatment with both test products increased *Prevotellaceae* levels, mainly in the PC. Saccharolytic fermentation by members of this bacterial family results in the production of acetate and succinate [49], with the latter being a substrate for succinate-converting, propionate producing micro-organisms such as *Bacteroides* and *Veillonella* species [50]. In the current study, an increased abundance of *Veillonellaceae* was observed upon treatment with POF, but not upon OFO supplementation. However, it might be that specific members of the *Veillonellaceae* family were stimulated upon OFO administration, while others reduced, resulting in the absence of effects at the family level. Finally, both test products increased the abundance of *Enterobacteriaceae*. This bacterial family contains several opportunistic pathogenic species, however, also many commensals able to ferment proteins are members of this bacterial group [51]. The strongest effects were observed upon OFO supplementation, which correlated with the more strongly increased levels of branched SCFA and ammonium, which are both markers of proteolysis that were observed upon fermentation of OFO as compared to POF. Overall, these results indicate that in vitro gut models combined with accurate molecular techniques have the potential to highlight specific microbial taxonomic changes and functional activities pathways upon dietary intervention. 

From this study, it can be concluded that the novel oat product POF and the commercially available OFO, when administered at similar β-glucan load, exerted equivalent prebiotic activity in the human gastrointestinal tract in vitro, with profound effects being observed on *Lactobacillus* and *Bifidobacterium* levels. The stimulation of *Lactobacillus* spp. observed in vitro was confirmed during an in vivo trial investigating the effect of OFO in human subjects with mild hypercholesterolemia. Moreover, the in vitro prebiotic activity of POF remained at lower β-glucan levels, demonstrating the potent prebiotic potential of the novel oat product even when administered at lower concentrations.

## 4. Materials and Methods 

### 4.1. Chemicals and Test Product

Unless otherwise stated, all chemicals were obtained from Sigma-Aldrich (Overijse, Belgium). PepsiCo, Inc. (Barrington, IL, USA) provided the different oat ingredients, including Quaker whole grain pre-cooked oat flour (POF) and old-fashioned oats (OFOs). OFOs were produced by steaming the whole groats to make them soft and pliable and then pressed to flatten them obtaining a particle size of 0.51−0.76 mm. POF was produced using PepsiCo’s proprietary process providing a ready-to-use format with improved dispersibility. The granulation range for POF ranged from 50–250 μm with the targeting of 178–250 μm. The daily dose of both oat ingredients during the in vitro experiment was standardised to provide 1.4g β-glucan once per day. For POF, two additional test doses were investigated—standardised to provide 0.3 g β-glucan twice per day (to total 0.6 g β-glucan per day) and 1.0 g β-glucan once per day. In order to mimic the in vivo conditions as close as possible, the test ingredient OFO was cooked according to the manufacturer’s instructions prior to use in the in vitro experiment. During the in vivo study, dry OFO was supplied to the subjects and cooked according to the manufacturer’s instructions prior to consumption. The consumption of cooked OFO was investigated during an intervention period of six weeks, providing a daily one serving dose of 40 g oats containing 1.4 g β-glucan. The control test product during the in vivo trial was Cream of Rice (Nabisco, East Hanover, NJ, USA), a cooked cereal containing no fibre or β-glucan and was supplemented at a dose of 40 g per day.

### 4.2. Simulator of the Human Intestinal Microbial Ecosystem (SHIME®)

The reactor setup simulating the human gastrointestinal tract was derived from the SHIME^®^ (ProDigest and Ghent University, Ghent, Belgium) as described by Molly et al. [23]. To optimally address the research questions, the SHIME^®^ setup was adapted from a single SHIME configuration (including one SHIME^®^ arm) to a TripleSHIME^®^ configuration (including three SHIME^®^ arms). Each arm of the TripleSHIME^®^ consisted of a succession of three reactors simulating the different regions of the gastrointestinal tract. The first reactor mimicked the upper gastrointestinal tract with the subsequent simulation of a gastric and small intestinal phase. The two subsequent colonic reactors simulated the proximal colon (PC), operated at pH 5.6–5.9 with a retention time of 20 h, and the distal colon (DC), operated at pH 6.6–6.9 with a retention time of 32 h. In order to simulate both the luminal and mucus-associated microbial community in the colonic reactors, mucin-covered beads were included as described by Van den Abbeele et al. [25]. Inoculum preparation, temperature settings, feeding regime, and reactor feed composition were adopted from Possemiers et al. [52]. To evaluate the properties of four different test ingredients and/or concentrations, four parallel TripleSHIME^®^ experiments were executed using the microbiota of three healthy adult human donors with elevated total and low-density lipoproteins (LDL) cholesterol levels (total ≥ 5.5 mmol/L and <7 mmol/L and LDL ≥ 3.4 mmol/L and ≤ 4.9 mmol/L) and a BMI between 20 kg/m^2^ and 25 kg/m^2^ (donor A: F, 25 y; donor B: F, 30 y; Donor C: M, 35 y), meaning that each test ingredient/concentration was tested in a separate TripleSHIME^®^ experiment including the microbial community of three different donors. Upon inoculation with the faecal inoculum from the different donors, a two-week stabilisation period was initiated allowing the faecal microbiota to differentiate in the colonic reactors depending on the local environmental conditions. Subsequently, the baseline microbial community composition and activity were determined in the PC and DC during a two-week control period, followed by an evaluation of the effects of repeated daily administration of the test products during a three-week treatment period. The test products were pre-digested prior to administration to the colonic reactors in order to produce relevant product fractions that would reach the colonic environment. Pre-digestion was performed as previously described by Van den Abbeele et al. [7], with some minor modifications. During the oral phase, the test ingredients were diluted to obtain a concentration mimicking the daily test doses of interest, corrected for moisture content. Furthermore, the intestinal dialysis approach included a 4.5-hour incubation, during which the dialysis fluid was replaced once every 45 minutes with fresh fluid. 

### 4.3. In vivo Study

This was a randomised, single-blind, placebo-controlled, and cross-over study to assess the effect of hot cooked OFO on a range of variables including faecal bacterial composition and plasma SCFAs, in a sample of healthy adults with elevated cholesterol levels within the same range as the in vitro study. There were six visits in total with two phases to data collection (Figure S1). Each phase consisted of a 10-week period. The trial phase comprised of a six-week intervention period (Week 0–Week 6) after which subjects completed a four-week washout (Week 10). 

In total, 34 randomised subjects (aged 18–65 years old, BMI of 18.5–30 kg/m^2^, fasting blood glucose (FBG) of 3.0–6.0 mmol/L) were included in the analysis. To enrol in the trial, subjects provided written informed consent, and then completed the required screening procedures to evaluate their eligibility for the trial. At screening (Visit 1), fasting blood samples were collected to examine potential subject’s safety and blood lipid profiles, the subjects were also administered a food frequency questionnaire (FFQ); this information determined eligibility. The study participants were required to have a low to moderate consumption of dietary fibre based on data from the 2008–2010 Irish National Adult Nutrition Survey [53] (Low- fibre diet: ≥9.9 g/d and ≤17 g/d for males; 8.2 g/d and ≤14.3 g/d for females; moderate extends to 25.1 g/d for males and to 22.3 g/d for females), and elevated total and LDL cholesterol levels (total cholesterol ≥ 5.5 and <7 mmol/L and LDL ≥ 3.4 mmol/L and ≤ 4.9 mmol/L). The trial included a two-week run-in period to washout possible pre-trial prebiotics prior to baseline assessment. Eligible subjects were then randomised in a 1:1 ratio at Visit 2 to receive either treatment or control product in Phase 1 (Baseline: Visit 2; Week 0—End of Phase 1: Visit 3 at Week 6; Washout period: Visit 4 at Week 10), and they would then cross-over to consume the alternative product for Phase 2 (Baseline: Visit 4 at Week 10; Week 10—End of Phase 2: Visit 5 at Week 16; Washout period: Visit 6 at Week 20). Group 1 received OFO during the first intervention period and Cream of Rice during the second intervention period, while Group 2 received Cream of Rice during the first intervention period and OFO during the second intervention period. Subjects were provided with a stool collection kit at visits 1, 2, 3, 4, and 5 and instructed to collect a sample at home (24 hours prior to their visit) and bring it to the clinic at their next visit. Samples were kept chilled preceding analysis.

The subjects consumed the study product once daily, at breakfast, for six weeks starting on Day 1, the day after Visit 2. The subjects were asked not to consume any other prebiotic/probiotics and fibre supplements or whole-grain oat products throughout the duration of the trial (22 weeks). They were also asked to maintain their habitual lifestyle in relation to physical activity level and diet.

Ethical approval was granted by the Clinical Research Ethics Committee, Cork Ireland, prior to the study starting with code ECM 5 (6) 17/01/18. The study was conducted according to the principles of ICH-GCP and the Declaration of Helsinki.

### 4.4. Microbial Metabolic Activity

During the in vitro SHIME^®^ experiment, samples for analysis of microbial metabolic activity were collected three times per week during the control and treatment period from each colonic reactor. Short-chain fatty acid (SCFA) measurements were performed as described by De Weirdt et al. [54] and included acetate, propionate, butyrate, and branched-chain fatty acids (isobutyrate, isovalerate, and isocaproate; bCFA). Lactate levels were determined using a commercially available enzymatic assay kit (R-Biopharm, Darmstadt, Germany) according to the manufacturer’s instructions. Ammonium determination was conducted as previously reported by Duysburgh et al. [55]. 

During the in vivo experiment, samples for analysis of plasma SCFA were collected at each scheduled visit, starting from Visit 2. Four plasma samples were collected in total per participant, i.e., one sample per baseline and endpoint of Phase 1 and Phase 2 of the in vivo experiment. Samples were prepared, extracted, and subjected to Ultra Performance Liquid Chromatography-Tandem Mass Spectrometry (UPLC-MS; Waters Ltd. Herts, United Kingdom) and analysed according to Brown et al. [56].

### 4.5. DNA Extraction

During the control and treatment period of the in vitro SHIME^®^ experiment, samples for microbial community analysis were collected once per week from each colonic reactor. Total DNA was isolated as described by Boon et al. [57], with some minor modifications as previously reported by Duysburgh et al. [55]. For the in vivo study, metagenomic DNA was extracted from all faecal samples by Teagasc (Cork, Ireland) using a modified version of the QIAGEN FAST Stool extraction kit (QIAGEN, Manchester, UK). The modification included an additional bead-beating step at the start of the procedure. Briefly, faeces were added to the buffer as described by the manufacturer’s instructions, after which homogenisation with a bead beater device was performed, followed by the protocol for Gram-positive bacteria as described by manufacturer’s instructions.

### 4.6. Microbial Community Analysis through qPCR

qPCR assays to quantify *Lactobacillus* spp. and *Bifidobacterium* spp. were completed using a QuantStudio 5 Real-Time PCR system (Applied Biosystems, Foster City, CA, USA). Each sample was analysed in technical triplicate and outliers (more than 1 C_T_ difference) were removed. The qPCR assay, including primer sequences and amplification program, for *Lactobacillus* spp. was conducted as reported by Furet et al. [58], while the qPCR for *Bifidobacterium* spp. was previously described by Rinttilä et al. [59].

### 4.7. Microbial Community Analysis through 16S-Targeted Illumina Sequencing

In order to compare effects on microbial community composition of treatment with OFO and POF at a dose of 1.4 g β-glucan per day, microbial community profiling of the luminal PC and DC compartment of the in vitro SHIME^®^ experiment was performed using 16S-targeted Illumina sequencing. Library preparation and sequencing on an Illumina MiSeq platform with v3 chemistry (2 × 300bp) were conducted by LGC Genomics GmbH (Berlin, Germany) using the primers as reported by Klindworth et al. [60], with modification of the reverse primer (785Rmod; 5’-GAC TAC HVG GGT ATC TAA KCC-3’) to increase coverage. 

### 4.8. Flow Cytometric Determination

Samples were also collected once per week from each colonic reactor for enumeration of bacterial cells via flow cytometry. A 10-fold dilution series was prepared initially in phosphate-buffered saline. Assessment of the viable, non-viable, and total population of the microbial community was done by staining the appropriate dilutions with SYTO 24 and propidium iodide. Samples were analysed on a BD FACSVerse (BD Biosciences, Erembodegem, Belgium). The samples were run using the high flow rate. Bacterial cells were separated from medium debris and signal noise by applying a threshold level of 200 on the SYTO channel. Proper parent and daughter gates were set to determine all populations. Results were obtained as log counts/mL.

### 4.9. Data and Statistical Analysis

For the analysis of 16S-targeted Illumina sequence data, read assembly and clean-up was adopted from the MiSeq procedure [61,62]. Briefly, mothur (v. 1.40.5, University of Michigan, Ann Arbor, MI, USA) was applied to assemble reads into contigs, perform alignment-based quality filtering (alignment to the mothur-reconstructed SILVA SEED alignment, v. 123), remove chimeras, assign taxonomy using a naïve Bayesian classifier [63] and SILVA NR v132 and cluster contigs into operational taxonomic units (OTUs) at 97% sequence similarity. All sequences classified as Archaea, Chloroplasts, Eukaryota, and Mitochondria were omitted, in addition to sequences that could not be classified at all. For each OTU, representative sequences were picked as the most abundant sequence within that OTU. To identify related bacterial species, the obtained sequences were matched using the SeqMatch tool in the Ribosomal Database Project software (Michigan State University, East Lansing, MI, USA) [64]. Results were presented as proportional values versus the total amount of sequences within each sample. Combining the proportional values of the 16S-targeted Illumina together with the enumeration of the total cell count obtained through flow cytometry, as was previously described by Vandeputte et al. [65], provided quantitative abundances of the different taxonomic entities inside the colonic reactors. These quantitative abundances were obtained by multiplying the proportional values of the 16S-targeted Illumina with the total log cell count obtained by flow cytometry. 

With respect to microbial parameters during the in vitro SHIME^®^ study, consistent findings were made for the three different donors in response to the treatment with the different oat products so that the averages of all parameters over the three donors were calculated per colon compartment for optimal visualisation of donor-independent treatment effects. Statistical analyses were carried out using Statistical Analysis Software version 9.4 (SAS Institute, Cary, NC, USA). Data were analysed by comparing the averages over the three donors for the control period as compared to the treatment period, and for the differences between the test product during the treatment period and this for both PC and DC separately, using Bonferroni correction. For 16S-targeted Illumina sequence data, statistical analysis was only performed for those bacterial families with complete data from at least two donors to capture both the noise within each donor and variability between donors. The significance level for all statistical tests was set at α = 0.05. Shapiro–Wilk tests were conducted to assess the normality of the data. Log-transformation was applied where appropriate.

For the in vivo study, outcomes were analysed on the per-protocol population (PP), i.e., all subjects with complete data and at least 80% compliance in terms of the amount of product consumed. Statistical analysis was performed using the SPSS Statistics software (IBM, Armonk, NY, USA), version 25. Normality of data and equality of the variances were determined with a Shapiro–Wilk test and a Levene’s test, respectively. For normally distributed data with equal variances, a one-way ANOVA with a Bonferroni post hoc test was used. For normally distributed data with unequal variances, a Welch test with a Games–Howell post hoc test was conducted. For non-normally distributed data, a Kruskal–Wallis one-way ANOVA test with multiple post hoc pairwise comparisons were performed. In terms of statistics, the differences indicated by ‘*p* < 0.05’ were significant. All data were normalised to the DNA concentration of the extracts obtained from the faecal samples.

## Figures and Tables

**Table 1:** Effects on microbial community composition as assessed through qPCR. The average increase in *Lactobacillus* and *Bifidobacterium* levels (16S rRNA gene copies/mL for lumen and 16S rRNA gene copies/g for mucus) during the treatment period as compared to the control period upon treatment with OFO at a dose of 1.4 g/d and POF at a dose of 0.6 g/d, 1.0 g/d, and 1.4 g/d in the luminal and mucosal environment of the proximal (PC) and distal colon (DC) of the human gastrointestinal tract for three human donors tested. For optimal observation of consistent effects over the different donors tested, the average of the three donors is presented (*n* = 3). The intensity of the shading correlates with the absolute abundance, which is normalised for each of the different bacterial groups in the different colonic environments, respectively. Statistically significant differences relative to the control period are indicated with underlining (*p* < 0.05). Statistically significant differences between the different test conditions within a colonic region are indicated with different letters (a, b, c), with conditions not sharing any similar letter being significantly different from each other (*p* < 0.05).

		PC								DC							
		POF 0.6 g/d		POF 1.0 g/d		POF 1.4 g/d		OFO 1.4 g/d		POF 0.6 g/d		POF 1.0 g/d		POF 1.4 g/d		OFO 1.4 g/d	
***Lactobacillus***	**Lumen**	3.5 × 10^8^	^a^	1.4 × 10^9^	^a^	6.2 × 10^9^	^b^	4.3 × 10^8^	^a^	1.5 × 10^8^	^a^	9.1 × 10^8^	^b,c^	5.6 × 10^9^	^b^	2.8 × 10^8^	^a,c^
	**Mucus**	2.4 × 10^9^	^a^	4.0 × 10^9^	^a,b^	3.3 × 10^9^	^b^	1.2 × 10^9^	^a,b^	6.0 × 10^7^	^a^	7.8 × 10^7^	^a,b^	1.3 × 10^9^	^b^	8.7 × 10^7^	^a^
***Bifidobacterium***	**Lumen**	4.5 × 10^9^	^a,b^	9.0 × 10^9^	^a^	4.5 × 10^9^	^b^	7.2 × 10^9^	^a^	1.4 × 10^9^	^a^	4.2 × 10^9^	^b^	4.3 × 10^9^	^b^	3.6 × 10^9^	^b^
	**Mucus**	5.3 × 10^9^	^a,b^	1.4 × 10^10^	^a^	9.2 × 10^9^	^b^	1.0 × 10^10^	^a^	2.3 × 10^8^		5.8 × 10^8^		1.1 × 10^9^		5.6 × 10^8^	

**Table 2:** Effects on microbial community composition at the family level in the proximal colon. Abundance (log counts/mL) of microbial families in the luminal environment of the proximal colon (PC) of the human gastrointestinal tract at the end of the control (C; *n* = 3/donor) and the treatment (TR; *n* = 3/donor) period upon treatment with OFO at a dose of 1.4 g/d and POF at a dose of 1.4 g/d for three human donors tested. For optimal observation of consistent effects over the different donors tested, the average of the three donors is presented (*n* = 3). The intensity of the shading correlates with the absolute abundance, which was normalised for each of the different families. Statistically significant differences relative to the control period are indicated in bold (*p* < 0.05). Statistically significant differences between the different test products (i.e., TR–POF versus TR–OFO) are indicated with different letters (a, b), with conditions not sharing a similar letter being significantly different from each other (*p* < 0.05).

		Proximal Colon			
		POF		OFO	
		C	TR	C	TR
**Actinobacteria**	***Bifidobacteriaceae***	6.83	**8.03**	6.94	**8.12**
	***Coriobacteriaceae***	<LOD	5.76	<LOD	5.10
**Bacteroidetes**	***Bacteroidaceae***	7.53	**6.01**	7.55	7.85
	***Muribaculaceae***	<LOD	<LOD	<LOD	4.37
	***Prevotellaceae***	5.04	7.65	4.73	7.12
	***Rikenellaceae***	4.32	4.20	4.34	5.41
	***Tannerellaceae***	5.64	4.67	5.92	6.58
**Firmicutes**	***Acidaminococcaceae***	6.53	5.36	5.20	7.25
	***Enterococcaceae***	3.36	5.34	3.53	7.53
	***Erysipelotrichaceae***	<LOD	4.11	4.64	4.72
	***Lachnospiraceae***	7.33	**5.86**	7.37	7.76
	***Lactobacillaceae***	3.45	7.78	3.51	6.32
	***Ruminococcaceae***	3.60	4.09	3.71	4.80
	***Veillonellaceae***	6.96	**7.93**	7.14	7.20
**Fusobacteria**	***Fusobacteriaceae***	<LOD	<LOD	4.21	4.89
**Proteobacteria**	***Burkholderiaceae***	5.15	**5.62**	5.62	**6.65**
	***Desulfovibrionaceae***	5.40	4.41	5.66	4.93
	***Enterobacteriaceae***	4.91	**5.99**	5.27	**7.50**
	***Pseudomonadaceae***	4.75	4.14	5.20	4.59
	***uncultured***	<LOD	<LOD	3.24	4.20
**Synergistetes**	***Synergistaceae***	4.59	3.88	3.81	4.70
**Verrucomicrobia**	***Akkermansiaceae***	<LOD	<LOD	3.61	3.94

**Table 3:** Effects on microbial community composition at the family level in the distal colon. Abundance (log counts/mL) of microbial families in the luminal environment of the distal colon (DC) of the human gastrointestinal tract at the end of the control (C; *n* = 3/donor) and the treatment (TR; *n* = 3/donor) period upon treatment with OFO at a dose of 1.4 g/d and POF at a dose of 1.4 g/d for three human donors tested. For optimal observation of consistent effects over the different donors tested, the average of the three donors is presented (*n* = 3). The intensity of the shading correlates with the absolute abundance, which was normalised for each of the different families. Statistically significant differences relative to the control period are indicated in bold (*p* < 0.05). Statistically significant differences between the different test products (i.e., TR–POF versus TR–OFO) are indicated with different letters (a, b), with conditions not sharing a similar letter being significantly different from each other (*p* < 0.05).

		Distal Colon			
		POF		OFO	
		C	TR	C	TR
**Actinobacteria**	***Bifidobacteriaceae***	7.39	**8.26**	7.45	**8.13**
	***Coriobacteriaceae***	5.61	5.31	5.66	4.93
**Bacteroidetes**	***Bacteroidaceae***	8.35	8.20	8.30	**8.04**
	***Muribaculaceae***	5.26	6.23	4.87	6.02
	***Prevotellaceae***	6.55	6.95	6.27	**7.22**
	***Rikenellaceae***	6.29	**5.74**	6.12	6.10
	***Tannerellaceae***	7.26	**7.84**	7.28	7.64
**Firmicutes**	***Acidaminococcaceae***	6.61	**7.22**	6.79	6.94
	***Enterococcaceae***	4.39	6.11	4.34	7.88
	***Erysipelotrichaceae***	5.64	4.86	5.65	<LOD
	***Lachnospiraceae***	8.23	8.28	8.16	8.27
	***Lactobacillaceae***	4.53	8.25	4.45	6.76
	***Ruminococcaceae***	6.64	**6.12**	6.74	**5.90**
	***Veillonellaceae***	7.37	**8.04**	7.34	7.36
**Fusobacteria**	***Fusobacteriaceae***	5.62	6.34	5.70	7.66
**Proteobacteria**	***Burkholderiaceae***	6.48	**6.73**	6.52	6.31
	***Desulfovibrionaceae***	6.58	6.79	6.76	6.94
	***Enterobacteriaceae***	5.42	**6.36**	5.45	**7.63**
	***Pseudomonadaceae***	6.57	**5.52**	6.44	6.48
	***uncultured***	5.89	6.51	6.05	6.33
**Synergistetes**	***Synergistaceae***	6.93	7.52	7.16	**7.27**
**Verrucomicrobia**	***Akkermansiaceae***	7.24	**7.67**	7.41	**7.77**

## Data Availability

Data available on request due to restrictions.

## Supplementary Materials

The following are available online at https://www.mdpi.com/2076-0817/10/2/235/s1, Figure S1: Cross-over study design.
