# Supplementary figures and images for: In vitro–in vivo Validation of Stimulatory Effect of Oat Ingredients on Lactobacilli
Source: Pathogens. 2021 Feb 19;10(2):235. doi: 10.3390/pathogens10020235 (PMC7922649; doi:10.3390/pathogens10020235)

## Supplementary Materials:

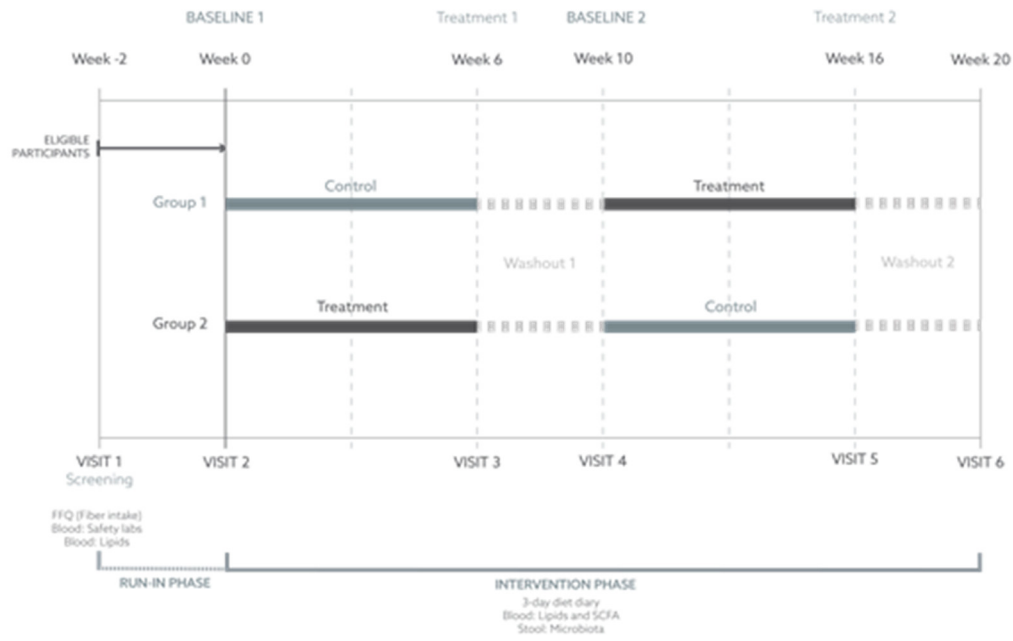

**Figure S1.** Cross-over study design.

Supplement: Supplementary file 1 [file pathogens-10-00235-s001.pdf]
